# A digital territory to be appropriated: the state and the nationalization of cyberspace

**DOI:** 10.12688/openreseurope.14010.2

**Published:** 2022-03-28

**Authors:** Thanos Koulos

**Affiliations:** 1 Public Administration and Sociology, Erasmus University Rotterdam, Rotterdam, South Holland, The Netherlands

**Keywords:** Nationalism, Cyberspace, National Identity, Nationalization of Space

## Abstract

Next to land, water, air and space, cyberspace is the complex socio-technical setting often called the ‘fifth domain’. Nationalism has taken over the organization of the first four domains, in the form of constructing national states, national territorial waters and national airspace. The basic proposition of this article is that the ideology of nationalism has also infiltrated the fifth domain – cyberspace – in two ways. First, through state-led cyber-nationalism via official government websites that present ‘national’ achievements and propagate the official state positions on disputes about territory, symbols or history. Second, through individual communities who use the internet to sustain a sense of national belonging and/or to promote and disseminate their nationalist ideals. Both ways are important in an online national identity (re)production framework that, in a fluid, global, modern world, functions supportively towards the traditional national identity (re)production mechanisms.

This article aims to examine the patterns of the nationalization of cyberspace through an analysis of state-led institutions and government websites that aim to enhance national identity and the sense of national belonging in a globalized world, as well as to propagate official state positions. It will focus on Greek, Dutch, US and Israeli websites. The term ‘nationalization’ in this context denotes the ideological charging of the cyber-footprint of the nation: how the internet produces and re-produces the nation, how the users partake in the national community by way of ‘consuming’ the digitalized national ideology, and the way cyber-nationalism defines people’s sense of belonging.

## Plain language summary

This article is part of a research project on the nationalization of cyberspace and nationalism-driven cyber-attacks, co-funded by MSCA and the Delft-Erasmus-Leiden consortium. The article examines the intrusion of the ideology of nationalism into the cyberspace, via the websites of the ministries of foreign affairs of four specific countries. The article finds that the official state national ideology is reflected online and propagated via the specific websites. This contradicts the original purpose of the Internet as a liberating, post-national medium, offering new forms of community. The main argument is that the ideology of nationalism has shaped our understanding of space – along ‘national’ lines – and this applies also to the cyberspace.

## Introduction

Following the establishment and evolution of modern nation-states since the 18 ^th^ century, our perceptions of space have been shaped by the ideology that defined the modern state system: nationalism. As a discursive formation shaping the modern world, nationalism could not have spared our modern perceptions of space and has managed to frame them in terms of its intrinsic qualities – that is ‘national’. Land was carved along ‘national’ states and territories, the air space turned to ‘national’ air space, while the seas became ‘national’ territorial waters and were subsequently carved into exclusive economic zones (of national states). Even the continental shelf was divided and became a property of the national state, to be exploited along with ‘national’ interests. International treaties and agreements regulated the delineation of these spaces. This process has been described by scholars as the ‘nationalization of space process’ and emphasis has been given to the construction of national homelands. After all, modern nation states, in their attempt to consolidate and exercise power and control over a territory, revert to practices of ‘nationalization’ of the specific territory where legitimacy is asserted through proof that the territory belongs to the specific nation. Despite the fact that emphasis has been given on the land component of space, we may argue that the nationalization of space process applies to air and sea spaces as well, transforming them to essential parts of the national space.

The past few decades, however, have witnessed the emergence of another type of ‘space’ – that is cyberspace. The rapid evolution of technology, and especially of the Internet, allowed the development of a decentralized nodal area that ‘links the nation across borders and oceans’ and a ‘new ecumene that enables many of the communicative, cultural and socioeconomic exchanges, which, in the previous 150 years, could only have existed within the structure of a nation-state’ ( Saunders, 2011). While the Internet ideology of the 1990s contained promises and hopes of a truly post-national realm, projecting the Internet as a liberating, empowering medium that offered new forms of community in bringing people together as citizens of a virtual global village, these proved utopian ( Sarikakis & Thussu, 2006). Nationalism managed to infiltrate the Internet, while cyberspace – or the ‘fifth’ domain – became a territory to-be-conquered. Although the modern state plays a major role in the formation, re-formation and expression of nationalist ideology, often nationalist ideology functions independently of the state or alongside it, especially when adopted by alternative, non-state-controlled institutions or individual groups. One may refer here to those nationalist ideologies and movements in eastern Europe for example, that strove to create their national states with the collapse of the empires in the region. The main aim of those nationalisms was the establishment of their own state, which once formed, took upon its institutionalized demeanour to re-produce the nation and its ideology throughout the times to come.

The main argument of this article is that the newly emerged cyberspace did not manage to spare itself from the power of the spell nationalism has cast upon our modern perceptions of space. This of course matches our (biased) way of thinking of all dimensions of social life along ‘national’ terms. Cyberspace turns thus into an often contested, non-territorial, digital ‘space’, carved up, claimed and conquered by nationalism. Every form of nationalism – whether connected to a nation-state of its own or not – has infiltrated the Internet and established a digital realm through which to promote its goal: to re-produce the nation and its ideology. Cyberspace is carved up along national lines, while national cyber-wars and cyber-defence practices are common new phenomena, often not under the full control of the nation-state. Given the power-grip of nationalism to our way of thinking, the nationalization of cyberspace seems to have been unavoidable. In this context, nationalism is defined as ‘an ideological movement for attaining and maintaining autonomy, unity and identity on behalf of a population deemed by some of its members to constitute an actual or potential nation’ ( Smith, 1991a). ‘Nation’ is defined as ‘a named human population sharing a historic territory, common myths and historical memories, a mass public culture, a common economy and common legal rights and duties for all members’ ( Smith, 1991a). To this useful definition, we shall include Walker Connor’s ‘sense of belonging’ ( Connor, 1978); an irrational psychological bond which supposedly constitutes the essence of national identity and binds fellow nationals together.

## Methodology

While the nationalization of cyberspace may not be attributed to a single actor, this article aims to examine the role of the official state – that is the encroachment of the official state-led nationalism into cyberspace and specifically, the reproduction of nationalism and national identities through governmental websites. It seems to have been imperative that every state institution be reflected online, propagating the institutionalized nationalist ideology through the Internet and in this way enhancing the digital national print, verifying in a sense the existence of the nation as a collectivity. As an examination of all state institutional websites would exceed the scope and limits of this article, the article will focus on the official websites of the ministries of foreign affairs of Greece, the Netherlands, the USA, and Israel. Greece and Israel were chosen as cases of eastern, ethnic types of nationalism, while the Netherlands and the US as western, liberal types. The official websites of the ministries of foreign affairs were selected as these institutions tend to more clearly reflect the dictates of the contemporary, state-led nationalist ideology.

The article will employ online content analysis and cyber-ethnography to analyze these websites along the general lines of the ethno-symbolist model on the nationalization of space and the construction of national homelands. Ethno-symbolism stresses the importance of myths, memories and symbols in the formation of nations and provides a model for the construction of national homelands as integral elements of national identities ( Smith, 1999; Smith, 2009). The English versions of the websites will be examined, as these are for external consumption and serve to articulate and delineate the cyber-spatial frontiers of the nation. All pages of the websites were scrutinized in order to trace the themes of nationalist ideologies and how they get reproduced online. Data collection involved note taking and taking screenshots of the relevant web pages.

### The nationalization of all spaces

 In the process of nationalizing the space, nationalism came to appropriate and alter our perceptions of all spaces along its terms. On land, nationalism found its main field of expression. As land provided the home of the nation, it had to turn into a national territory. This process was multi-dimensional and primarily symbolic, as symbols have the capacity to generate collective emotions and feelings of belonging on the one hand, while demonstrating to ‘outsiders’ to whom the particular territory belongs to, on the other. Language, as the most symbolic form of communication, has been the primary mode of the nationalization of space; from the naming (and re-naming) of towns, streets, squares, natural features and everything associated with the collective life, language is one of the most distinguishing ‘national’ characteristics that is effortlessly utilized to nationalize the territory. At the same time, national monuments and symbols are employed in order to ‘mark’ national space as representations of a specific national past.

This process becomes more obvious in cases of territories that change national ‘hands’ usually after a conflict. Of the most radical and palpable nationalization of space processes has been the one undertaken by Turkey and the Turkish Cypriots in the northern part of Cyprus since 1974 and the division of the island. From carving flags into the mountains, changing the names of all towns, villages, streets, mountains, creeks, rivers and destroying or seriously undermining Greek Orthodox Christian monuments in favour of – mostly new – mosques, to introducing Anatolian populations as well as statues of prominent Turkish figures (primarily of Kemal Ataturk) and Turkish flags to every town and village square, the campaign to ‘Turkify’ ^1^ northern Cyprus has been systematic. This again has had a two-fold message: one for internal consumption – that is to galvanize a sense of ‘Turkishness’ of the land to Turkish Cypriots and formulate a connection between their national identity and that particular stretch of territory, and a message to outsiders – primarily the Greek Cypriots – that this land is now Turkish ( Koulos, 2018; Koulos, 2021). Along similar lines, the intense ideological clash of another two contradictory and exclusive national narratives in neighbouring Palestine and the victory of Israel, has led to a process of ‘Judaization’ ^2^ of Palestine and the undermining of Palestinian presence from space and time ( Zertal & Eldar, 2009; Zreik, 2016). Since the 1948 victory of Israel, land development practices have aimed to provide material reality to the historical narratives and values of the new Hebrew state, leaving no room for the Arab presence ( Aron, 2019).

With regards to the maritime space, coastal nation-states have turned this to ‘national’ space, as a natural extension of their territory. International treaties and agreements came to delineate and regulate the control of the nation in its maritime space. The United Nations Convention on the Law of the Sea (UNCLOS), or the ‘Law of the Sea Convention’ is an international agreement that established guidelines for the management of marine natural resources, the environment and businesses, defining the rights and responsibilities of nations with regards to their use of the oceans. ^3^ The 1982 convention replaced previous treaties and came into force in 1994. Of interest for this article is the choice of names that many nation-states have given to natural gas and oil reserves that have been discovered in their continental shelf. Adhering to the process of the nationalization of space, many nation-states choose symbolic names that stem from their mythology and tradition. This is to verify the ownership of the natural resource and the maritime space in symbolic terms and ‘mark’ it as national – as rightfully belonging to the nation since the continental shelf is a natural extension of the ancestral homeland. Examples are the reserve ‘Aphrodite’ in the Cypriot continental shelf, as well as the reserves ‘Leviathan’ and ‘Tamar’ in the Israeli continental shelf ( Koulos, 2018). The symbolic nationalization of maritime space is one aspect of ‘resource nationalism’, that is the assertion of control by a state’s government over natural resources located on their territory ( Koch & Perreault, 2019). This further points to the inability of nationalism to tackle global issues, like biodiversity loss. Regarding airspace, this has again turned to ‘national’ airspace as nation-states have complete and exclusive sovereignty over the airspace above their territory, including above their territorial waters. The Convention on International Civil Aviation established the International Civil Aviation Organization – a UN specialized agency for coordinating international air travel. ^4^ This Convention further established the rules with regards to airspace and state jurisdiction. 

Furthermore, there are also cases where the nationalization of space continues even if the territory is lost for the nation. These are the cases of the ‘lost homelands’. Many nations have lost parts of their perceived ancestral homeland, usually after military defeats and population transfers, but the nationalist ideology continues to regard them as ancestral homelands perpetuating their nationalization by turning them to *lost* national homelands. In these cases, the nationalization of the lost space is purely conceptual and imaginary, since there is no actual space to become nationalized. Examples of lost homelands may be found in the national narratives of the Armenians, the Greeks, the Germans and the Serbs among others ( Koulos, 2016).

Accordingly, the emergence of another type of ‘space’ could not have gone unnoticed and not become interpreted along national terms. Cyberspace describes a psycho-geographic environment, the abstract nature of which makes it difficult to define ( Kitchin, 1998). The term ‘cyberspace’ was first introduced by William Gibson in his novel *Neuromancer* and is defined as a ‘consensual hallucination experienced daily by billions of legitimate operators, in every nation […] the graphical representation of data abstracted from the banks of every computer in the human system’ ( Gibson, 1984). Michael Benedikt expands Gibson’s innovative concept by describing cyberspace as a new, parallel universe that is ‘created by the world’s computers and communication lines’, sustained by a ‘common mental geography, built by consensus and revolution, canon and experiment’ and connected by corridors forming ‘wherever electricity runs with intelligence’ ( Benedikt, 1992). Jim Falk argues that browsing the web results to ‘experience movement over an information terrain, mapped not by geography solely or even particularly, but by a multidimensional set of categories and themes’ ( Falk, 1998). Ronald Deibert defines cyberspace as ‘the artificial “space” one enters on computer networks’ ( Deibert, 1997), while Saskia Sassen argues that the digital space of the Internet is divided between private and public realms – like the territorial space – something that results to distinctions in communication, access and content in cyberspace ( Sassen, 2000). Finally, some perceive cyberspace to be a new electronic frontier built to be colonized solely by the rich, white and powerful ( Sardar, 1998). For the purposes of this article, Robert Saunders’ definition of cyberspace is adopted, as the ‘conceptual universe created by and sustained through electronic interactions of humans over global computer networks and shaped by ever-changing geographies of digitized information’ ( Saunders, 2011).

Cyberspace has allowed extraordinary prospects for nationalism, exactly because of its functionality. It has offered an alternative universe with infinite potential for the storage, preservation and reproduction of national identity symbols – like legends, folk songs, anthems, genealogies, art, pictures, contested versions of history, maps, alternative linguistic and orthographic forms, dialects, etc. – and has provided for the ultimate public sphere ( Nelson, 1996). Its deterritorialized nature releases the imaginative powers of nation-building, which do not depend on the physical proximity of the community’s members ( Candan & Hunger, 2008). The Internet provides national communities with ‘mass-mediated imaginaries, that is, spaces of memory ( *lieux de memoir*) physically tethered to real world geography, but free of many – if not all – of its restrictions’ ( Saunders, 2011). It further supports the creation of a Habermasian transnational public sphere where marginalized groups can ‘produce and debate narratives of history, culture, democracy and identity’ ( Bernal, 2006). Minorities and other national groups make use of the ability provided by the Internet to post web pages, web logs (blogs) and links to other sites, providing open, incendiary and even exaggerated or completely false information about their situation. These groups may use the Web to inform the outside world of their economic, political and social condition, to articulate their political goals, demand independence or lobby for greater rights. Among the many layers of Cyberspace are autonomous forces, like diasporas that often become radicalized – especially when facing cultural assimilation by the host society – and use cyberspace to promote hostility, aggressive patriotism, xenophobia, and often, war ( Conversi, 2012). The role of Facebook has also been significant in inciting or assisting radicalization and nationalism. All this point to the extent of intrusion of nationalist ideology in cyberspace. The state, however, has not been absent from this nationalization of cyberspace.

Some argue that the nationalization of cyberspace was initiated in the mid-1980s with the introduction of geographic determinates for sites located outside the US. These sites came to include domain names which contained their country of origin at the terminus of the universal resource locator (URL) – for example .fr (France), .jp (Japan), etc. Internet use thus created a new social space that existed outside the limits of geographic space but was simultaneously rooted in ‘real-world spatial fixity’ ( Kitchin, 1998). Although the Internet provides the means to circumvent traditional state sovereignty, enabling its users to access diverse views and has made national boundaries more permeable ( Lengel & Murphy, 2001), the digital space is somewhat embedded in ‘actual societal structures and power dynamics: its topography weaves in and out of non-electronic space’ ( Sassen, 2000). The digital terrain of cyberspace may still be somehow shaped by national borders ( Halavais, 2000), but considering cyberspace as a geography it becomes apparent that nation-states are seriously underrepresented in virtual versus real space ( Saunders, 2011). Moreover, the concept of cyberspace includes 3 layers: physical, logical and cyber-persona layers. The physical layer refers to the machine infrastructure – computers, routers, cables, servers, cell phone towers, and satellites – that establish the mechanical and electrical, magnetic, and optical lines of communication. The logical layer refers to the logical instructions and software that operate communications traffic, such as domain name system (DNS) and Internet service providers (ISP). Finally, cyber-persona or social layer refers to the sphere through which videos, images, sounds, and texts circulate. This paper deals with the social layer and the messages articulated through the state websites. But let’s see how nation-states partake in the online national identity reproduction framework.

## State-led cyber-nationalization

Following the evolution of technology in the past 30 years, all nation-states have extended their institutional presence to the digital domain. Thus, all nation-state defining institutions also have an online presence, ‘drawing’ in a sense the digital boundaries of their national cyberspace. The most advanced nation-states have transcended some of their traditional functions by allowing the online execution of some of the transactions between their administration and the citizenry (i.e., e-government functions, online tax form submissions, online issuing of certificates, etc.). Of importance however for the nationalization of cyberspace has been the move to the digital domain of the nation-state institutions, since every nation-state defining institution has its own website. This article focuses on the websites of the ministries of foreign affairs of four countries: Greece, the Netherlands, the USA and Israel. The role of the ministry of foreign affairs is quite important in modern nation-states, since it promotes the national interests and positions of the country, articulating at the same time the current trends of the official nationalist ideology. As the ministry of foreign affairs represents the nation-state in the outside world, maintaining international relations and promoting its interests, the same way it represents it in the cyberspace, delineating in a sense the ‘national’ cyberspace. At the same time, we may argue that these websites digitally reproduce intrinsic characteristics of each nation.

### Greece

The website of the Greek ministry of foreign affairs is organized along the themes of ‘The Ministry’, ‘Foreign Policy’, ‘Current Affairs’, ‘Services’ and ‘Contact’. ^5^ The site is available in Greek, English and French. Its home page offers links to the latest announcements, statements and speeches of ministry officials. Under ‘Mission and Competences’, the website states that the ministry of foreign affairs ‘conducts the country’s foreign policy, represents the country before other states and international organizations, participates on its behalf in international cooperation initiatives and mechanisms at the international, European and regional levels and advocates Greek interests, both public and private, abroad’. ^6^ Regarding the competences of the ministry, the site states that they involve

‘ *representing Greece before foreign states […] enhancing the image of Greece and of Greek culture abroad and promoting international cultural cooperation; informing the international community about the possibilities for economic and business cooperation with Greece; safeguarding the rights and interests of Greek citizens abroad and providing assistance with their administrative affairs; fostering closer relations between the Greek Diaspora and their homeland, and supporting them in preserving their mother tongue and their distinctive cultural identity […]*’. ^7^

We thus see in this section the general guidelines on the role of the Greek ministry of foreign affairs, as well as some more subtle elements that touch upon Greek national identity and its representation and reproduction online. Greek national identity has a long-lasting fixation with the past and the alleged grandeur of ancient classical civilization and culture. Hence, one of the ‘competencies’ of the ministry is to ‘enhance the image of Greece and of Greek culture abroad’, exactly because Greek culture is perceived as one of the most distinguishing national characteristics. Related to this, and to the reproduction of Greek national identity, is the ‘competency’ of the ministry to ‘foster closer relations between the Greek Diaspora and its homeland’ and to support them in preserving their ‘mother tongue and distinctive cultural identity’. This demonstrates the dictates of Greek nationalist ideology to reproduce Greek national identity – through the basic institutionalized ‘weapons’ of language and culture – to the diaspora so as to ensure their ethno-cultural – if not civic – allegiance to the Greek nation.

An interesting element is that the digital avatar of the Greek ministry of foreign affairs includes a list with the original texts of all the major international treaties concerning Greece. ^8^ This is a list with 11 treaties that dealt with the territorial expansion of Greece, from the establishment of the Greek Kingdom with the 1830 London Protocol, to the 1947 Peace Treaty with Italy and the annexation of the Dodecanese islands by Greece. The incorporation of these treaties into the website of the Greek ministry is indicative of the efforts to validate the territorial integrity of the Greek state online, providing the legal framework that authenticates Greek territorial expansion and, in a sense, symbolically demarcating the Greek cyberspace with ‘proof’ and legal documents of the real space.

 Under ‘Foreign Policy Issues’, three issues figure prominently as ‘national’ ones – defined as such by Greek nationalist ideology of the past few decades: relations with Turkey, the Cyprus issue and the issue of the name of North Macedonia. ^9^ The other ‘issues’ – energy, cultural, religious, sports diplomacy and development cooperation – although depicted from a Greek national point of view, are ‘softer’ in the sense that they do not immediately touch upon sensitivities of national identity and security like the first three. Regarding Greek-Turkish relations, the website provides an overview of the issues between the two countries over the past 50 years, arguing that since the 1970s, Turkey ‘initiated a systematic policy of contentions and claims against the sovereignty, the sovereign rights and jurisdictions of Greece. The goal of this newly formed policy against Greece has been the changing of the territorial status quo provided for in international treaties – the Treaty of Lausanne being pivotal among these – and the legal status of maritime zones and airspace as they derive from international law and the law of the sea’. ^10^ With Turkey being the significant ‘other’ of Greek nationalism, and a stable variable in the formulations of Greek nationalist ideology and its perceptions of history, destiny and security, the website is representative of this dynamic relationship. Turkey is still portrayed as the ‘enemy’, a power that threatens Greek territorial integrity and security, with ‘illegitimate’ claims and actions, while Greece ‘is making an ongoing effort to convert Greek-Turkish relations from relations of conflict into relations of cooperation. That is why Greece is extending a hand of friendship to Turkey, calling on the latter to cooperate – in the spirit of consensus and constructiveness befitting neighbours – on improving Greek-Turkish relations and ironing out tensions’. ^11^ To further support this narrative, this webpage has a link to ‘Relevant Documents’, that explain in detail the Greek positions on the various issues between the two countries. ^12^ The pattern however remains the same: ‘good’, ‘honourable’, ‘democratic’, ‘European’ and – of course – ‘righteous’ Greece is threatened by the ‘authoritarian’, ‘barbaric’, ‘disrespectful’ and ‘greedy’ Turkey. Playing the card of the victim, apart from drawing attention away from your own responsibilities and obligations, functions cohesively for the group, strengthening their self-perception as a collectivity, a distinctive nation. Turkey has long played the role of the ‘wolf’ in Greek nationalist narrative, keeping the ‘herd’ hurdled together.

The second ‘national’ issue, related to the first, is the Cyprus Issue. The website provides an account of the Cyprus Issue from the 1974 Turkish invasion and occupation, to the various talks and negotiations and the Greek positions. It is an account of the Greek – and Greek-Cypriot – perspective, conveniently disregarding the Turkish – and Turkish-Cypriot – viewpoints, as well as the facts that led to the 1974 Turkish invasion – which the Turkish narrative emphasizes ( Papadakis, 2003). There is also a link to ‘Relevant Documents’, ^13^ a list of links to human rights and fundamental freedoms violations, UN resolutions on the Cyprus Issue, the inter-communal negotiations, and in general, documentation once again on the ‘righteousness’ of the Greek and Greek-Cypriot side (and who the ‘wolf’ is).

The third issue is the one of the naming of North Macedonia. The website provides an account of the issue, its background and its recent resolution, always from a Greek perspective. It argues that the term ‘”Macedonia” refers to the Kingdom and the culture of the Ancient Macedonians, who were part of the Greek nation, and constitutes an indisputable part of the historical and cultural heritage of Greece’. ^14^ We notice at this point a claim that sits at the core of the study of nationalism – that is the issue of the antiquity of nations, or the notorious question: when is a nation ( Connor, 1990)? The website claims that there existed a Greek nation in antiquity, expressing a primordialist approach of Greek national ideology. Primordialism holds that nations are natural and have existed since antiquity, disregarding modern factors that contributed to nation formation – like industrialism, capitalism, print technology, the development of the bureaucratic state, etc. ^15^ The website further argues that ‘the former Yugoslav Republic of Macedonia proclaimed its independence in 1991 by predicating its existence as an independent state on the artificial concept of the "Macedonian nation". Greece reacted strongly to the usurpation of its historical and cultural heritage and to the creeping territorial and irredentist claims of the new then country…’; ^16^ this points to the heart of the issue. The ancient Macedonian heritage and legacy was first appropriated by Greek national ideology, placing it within the Greek mythology and heritage from which modern Greek national identity was crafted. As a result, any alternative narrative – that for example the Macedonians might have been a separate nation – was seen as heretic and challenging to the established Greek national identity. Hence the intense reactions from Greece at the use of the name ‘Macedonia’ by the neighbouring country, that even today this website articulates with the ‘artificial concept of the “Macedonian nation”’. By considering the concept of the ‘Macedonian nation’ artificial, the website – and by definition, Greek national ideology – undermines one of the fundamental principles of national self-determination of the neighbouring country – that is the right to freely choose their collective name ( Koulos, 2016). The website further cites the final agreement on the name dispute between the two countries – the Prespa Agreement – signed in 2018. ^17^ Using the leverage of NATO and EU participation, Greece managed to achieve the constitutional name change of the neighbouring country to ‘North Macedonia’ (from ‘Macedonia’), as well as to preserve its monopoly over the ancient Macedonian legacy. The treaty explicitly states that the two Parties ‘note that the official language and other characteristics of the Second Party (of North Macedonia) bear no relation to the ancient Greek culture, to the history, culture and heritage of the northern region of the First Party (of Greece)’. ^18^ In this way, the website of the Greek ministry of foreign affairs marks the cyber-spatial boundaries of Greece and its perceived national cultural heritage, with the ancient Macedonian legacy on the Greek side of the ‘border’, ‘officializing’ it by citing the Prespa Agreement which clarifies the sole ‘ownership’ of ancient Macedonia by Greece.

### The Netherlands

The website of the Dutch ministry of foreign affairs is organized along the themes of ‘Organization’, ‘Contact’, ‘Events’, ‘Diplomatic Statements’, ‘The BZ Story’, ‘Privacy’, ‘Knowledge and Research’, ‘The Work of BZ in Practice’, ‘News’, ‘Agenda’, ‘Subjects’, ‘Documents’, ‘Information for the Press’ and ‘Travel Advice’. The site is available in Dutch, English, Papiamento and the Dutch sign language, while its home page offers links to featured announcements and news. ^19^ Under ‘Organization’ the site states that

‘ *[…] The Ministry of Foreign Affairs is committed to Dutch interests and values worldwide. Our embassies in some 150 countries act as both the antenna and the mouthpiece of the Netherlands. We help Dutch people abroad and we negotiate and cooperate in many areas in the European Union and within organizations such as NATO and the United Nations*’. ^20^

Under ‘the BZ Story’, the site argues that the ministry is working for a) safety ‘in the Netherlands, Europe and further afield’, b) welfare, since ‘due to exports and investments our income largely comes from abroad’, c) a fair and sustainable world as ‘global challenges require global cooperation and prevention’, and d) to support Dutch people abroad. ^21^

The website of the Dutch ministry of foreign affairs is representative of the general outlook of the Dutch national identity. Outward-looking, civic, with a touch of cosmopolitanism, grounded in the political reality of the present without much sentimental affection for the past (unlike the Greek national identity), Dutchness feels almost anti-national. Isn’t this anti-national element however perceived to be a constitutive and positive feature of the Dutch ( Beyen, 2008)? Many claim that anti-nationalism is a politicised and informal form of Dutch nationalism, with a long history in the Netherlands ( Kesic & Duyvendak, 2016). In this respect, the anti-national character of the website of the Dutch ministry of foreign affairs seems to follow and digitally reproduce this Dutch pattern of anti-nationalism. The website further portrays an image of tolerance, open-mindedness, pluralism and progress – elements again of what the Dutch national identity aspires to distinguish itself with. These are after all central elements in the historicized image of Dutchness ( Van Reekum, 2012).

Finally, the website provides detailed policy and operations evaluations, as well as evaluations of foreign policy spending. This is to ‘identify the results achieved, thus giving an indication of effectiveness and efficiency, as well as the factors that led to these results’. ^22^ This may be perceived as quite corporatist, but a high degree of corporatism is another particular trait of the Dutch national identity ( Kesic & Duyvendak, 2016). We thus notice that the website of the Dutch ministry of foreign affairs follows the dictates of current Dutch nationalism, by digitally reproducing the Dutch national identity’s characteristics. In this way, the site ‘stamps’ as distinctively Dutch the cyber-spatial dimension of the Netherlands.

### The USA

The US Department of State website is mapped along ‘Policy Issues’, ‘Countries and Areas’, ‘About’ and ‘Bureaus and Offices’. It is available only in English and it has links to ‘Press’, ‘Business’, ‘Employees’, ‘Job Seekers’, ‘Students’ and ‘Travelers’. ^23^ Under ‘Vision’ the site states that ‘on behalf of the American people we promote and demonstrate democratic values and advance a free, peaceful, and prosperous world’, while under ‘Mission’ it states that ‘The U.S. Department of State leads America’s foreign policy through diplomacy, advocacy, and assistance by advancing the interests of the American people, their safety and economic prosperity’. ^24^

American nationalism has been shaped by distinctive historical circumstances and has contributed to the development of a set of liberal, universal political ideals and a perceived accountability to propagate those principles worldwide. Recognizing the fact that the US is in a sense ‘accountable’ for spreading democracy and liberal change throughout the world has historically dictated the US foreign policy. This should be also seen with regards to another essential attribute of American nationalism, that is a sense of mission ( Grant, 2020). This sense of mission legitimizes the equation of US interests with those of humanity in general, something which in turn forms US global posture ( McCartney, 2002). Thus, the promotion of democracy should not be seen as simply another measure of US foreign policy, but rather as a fundamental characteristic of American national identity ( Monten, 2005). This may be considered a form of American exceptionalism.

 American exceptionalism is an ideology perceiving the history of the US as fundamentally different from that of other nations ( Lipset, 1996). This perception is based on a) the American Revolution origins of the US, b) that the US became the first ‘new nation’ ( Lipset, 1963), c) the development of ‘Americanism’ – that is a unique ideology based on liberty, equality before the law, individual responsibility, republicanism, representative democracy, and laissez-faire economics ( Lipset, 1996). Advocates of this ideology trace the exceptionality of the US to the fact that if was not founded on the grounds of common ethnicity, heritage or of a ruling elite, but on a set of liberal ideals. In his Gettysburg Address President Abraham Lincoln argued that America is a nation ‘conceived in liberty, and dedicated to the proposition that all men are created equal’ ( Williams, 1953). America is inextricably tied to equality and freedom, according to Lincoln, while its mission is to ensure that ‘government of the people, by the people, for the people, shall not perish from the earth’. Lincoln further believed that

*‘In the United States man would create a society that would be the best and the happiest in the world […] However, the Union did not exist just to make men free in America. It had an even greater mission—to make them free everywhere. By the mere force of its example, America would bring democracy to an undemocratic world’* ( Williams, 1953).

In order to advance democracy globally through its foreign policy, the US State Department has established an Undersecretary for Civilian Security, Democracy, and Human Rights, with the mission to ‘prevent and counter threats to civilian security. The bureaus and offices reporting to the Under Secretary advance the security of the American people by assisting countries around the world to build more democratic, secure, stable, and just societies’. ^25^ Furthermore, under this undersecretary there is a specific Bureau of Democracy, Human Rights and Labor. The mission of this bureau, as stated on the website is to

‘ *champion American values, including the rule of law and individual rights, that promote strong, stable, prosperous, and sovereign states. We advance American security in the struggle against authoritarianism and terrorism when we stand for the freedoms of religion, speech, and the press, and the rights of people to assemble peaceably and to petition their government for a redress of grievances*’. ^26^

‘Human rights and democracy’ are also noted as one of the ‘Policy Issues’ of the Department of State website, stating that

‘ *The protection of fundamental human rights was a foundation stone in the establishment of the United States over 200 years ago. Since then, a central goal of U.S. foreign policy has been the promotion of respect for human rights, as embodied in the Universal Declaration of Human Rights. Supporting democracy not only promotes such fundamental American values as religious freedom and worker rights, but also helps create a more secure, stable, and prosperous global arena in which the United States can advance its national interests. In addition, democracy is the one national interest that helps to secure all the others. Democratically governed nations are more likely to secure the peace, deter aggression, expand open markets, promote economic development, protect American citizens, combat international terrorism and crime, uphold human and worker rights, avoid humanitarian crises and refugee flows, improve the global environment, and protect human health…*’ ^27^

This illustrates the importance of democracy and its promotion for American national ideology and identity. Democracy is perceived as the single utmost national interest of the US, the one that secures all others. The US is bound to promote human rights and democracy not out of altruism or genuine concern over other people, but because this would create the environment necessary for the US to advance its interests. Democracy and human rights are thus instrumentalized by American national ideology, becoming constitutive elements of what it means to be American.

 Another peculiarity of the US Department of State and its website that reflects on issues of the American national identity, is the ‘Office of the Historian’. The Office of the Historian

*‘is responsible, under law, for the preparation and publication of the official documentary history of U.S. foreign policy in the Foreign Relations of the United States series. In addition, the Office prepares policy-supportive historical studies for Department principals and other agencies. These studies provide essential background information, evaluate how and why policies evolved, identify precedents, and derive lessons learned. Department officers rely on institutional memory, collective wisdom, and personal experience to make decisions; rigorous historical analysis can sharpen, focus, and inform their choices…’* ^28^

This Office of the Historian seems to have as its main goal the institutionalization of the foreign policy and foreign relations history of the US. In this way, through an institutionalized historical inquiry over its foreign relations, a sense of historicity is constructed for the relatively new American nation. It is an attempt to construct historical depth and continuity – something missing from American national identity. While other nations may claim historical depth both online and offline – like the Greek nation and the website of the Greek ministry of foreign affairs claiming that ancient Macedonians were part of the Greek nation, thus assuming the existence of a Greek nation and establishing its continuity since antiquity – the American nation is generally accepted that it is a ‘new’ nation. Its relative ‘youthfulness’ however does not mean that it does not need the anchoring and sense of mission that historical depth and continuity seem to provide to national identities.

We thus notice that the website of the US Department of State digitally reproduces elements specific to the American national identity – like the promotion of democracy and the institutionalization of history attempts in order to construct historical depth – delineating the cyber dimension of the American nation. This way, current American national ideology ‘Americanizes’ the US cyberspace through the State Department digital avatar.

### Israel

The website of the Israeli ministry of foreign affairs is organized along ‘MFA’, ‘Press Room’, ‘About the Ministry’, ‘Consular Services’, ‘Foreign Policy’, ‘International Orgs’, ‘About Israel’ and ‘Diplomacy in Video’, and it is available in English, Arabic, Hebrew, Persian, Russian and Spanish. ^29^ Under ‘About the Ministry’ the functions of the ministry are summarized accordingly:

- *‘The Foreign Ministry formulates, implements and presents the foreign policy of the Government of Israel*.
- *It represents the state vis-a-vis foreign governments and international organizations, explains its positions and problems throughout the world, endeavors to promote its economic, cultural, and scientific relations, and fosters cooperation with developing countries. Israel currently maintains diplomatic relations with 162 countries*.
- *The Ministry promotes relations with Diaspora communities and safeguards the rights of Israeli citizens abroad...’* ^30^

So, apart from formulating, implementing and presenting the Israeli Government foreign policy, the ministry is also charged with ‘explaining’ its positions and problems throughout the world. As we will further see in analyzing the website, what the ministry represents and ‘explains’ is the Israeli national viewpoint on the various issues – and specifically the Israeli-Palestinian conflict – thus advancing the current Israeli national ideology, one way being through this website.

 Importantly for this article, parts of the ‘About the Ministry’ section are also the ‘Database of Treaties’, the ‘Status of Relations’ and the ‘MFA History’. Regarding the ‘Database of Treaties’, the website contains links to all bilateral and multilateral agreements of Israel. In this way, a relatively newly established state that is not recognized by many (Arab) countries, and whose existence is systematically threatened by other countries (e.g., Iran) as well as terrorist organizations (e.g., Hamas) attempts to digitally verify and legitimize its existence. Through digitally reproducing the legal treaty documents Israel has signed and entered as equal to other states, the website reaffirms the existence of Israel as a sovereign state, and consequently of the Jewish nation that Israel was established to protect. Relevant to this is the ‘Status of Relations’ link of the website, which provides detailed information on the countries that have recognized Israel, the countries that do not recognize it, as well as the countries that maintained relations with it in the past but have since severed them. ^31^ The webpage provides a visual map, as well as a list of the countries with the date that they established relations with Israel. Again, this webpage tries to reiterate that Israel exists as equal to other states, with which it has diplomatic relations. The fact that this is reproduced on the website of the ministry also comes to affirm the cyber-existence of Israel, delineating its national cyberspace. This seems to confirm the argument that the more the nation sees dangers in its existence, the more it needs to legitimize it ( Abulof, 2014). Regarding the history of the ministry, the site contains links to all historical documents concerning Israel’s foreign relations. ^32^ Similarly to the US website, the Israeli website attempts in this way to institutionalize historical inquiry into its foreign relations, and to construct a sense of continuity and historicity between its modern institutions and the land. This continuity of the modern institutions comes to supplement the Jewish national narrative that claims the land of Israel for the Jews only, the main reason being an ‘unbroken chain of 5000 years of history’. ^33^

 Under ‘Foreign Policy’ the site offers links to ‘Operation Protective Edge’, ‘MFA Social Media Wall’, ‘Antisemitism Today’, ‘Behind the Headlines’, ‘Bilateral Relations’, ‘FAQ’, ‘Historical Documents’, ‘Worldwide Aid’, ‘Iran’, ‘Peace’, ‘Terrorism’, and ‘Legal Issues’. ^34^ So, if the order of the subjects under ‘Foreign Policy’ signifies the relevant importance given to them by the Israeli state, it could be interpreted that the ministry of foreign affairs is more occupied with justifying the state’s actions against Palestinians and doing ‘damage control’ in the international arena in support of these actions – thus the ‘explaining the Israeli positions’ function. Under ‘Operation Protective Edge’, the site provides further links to articles that support its 2014 military operation against the Gaza Strip. The operation came as a response to increasing rocket launches from the Strip to Israel but many characterized it ‘disproportionate’ with Amnesty International condemning it. ^35^ The website, in line with Israeli national positioning, fervently supports the operation and rejects any criticism of its actions. Some excerpts from the various links of this page are illustrative: ‘ *The methodology the report is based upon is fundamentally flawed, evidence that Amnesty has a flawed understanding of international law, and further reveals the organization’s compulsive obsessiveness towards Israel*’ (Israeli response to Amnesty International report, July 29, 2015); ‘ *It is regrettable that the report fails to recognize the profound difference between Israel’s moral behavior during Operation Protective Edge and the terror organizations it confronted*’ (Israeli response to the UNHRC Commission of Inquiry, June 22, 2015), etc. ^36^ There are about one hundred links to similar articles that support operation edge on this webpage alone, tuned along the same ‘frequency’ – of Jewish moral superiority and ‘righteousness’ of their cause, with no mention of the dead of the ‘other’ side. This element of moral superiority is inextricably linked to ethnocentrism in general, as well as to the Jewish beliefs of ‘choseness’ ( Smith, 2003), and of messianic redemption of the land ( Segev, 2007; Tekiner, 1991) in particular.

 The webpage of ‘Foreign Policy’ offers a link to ‘Iran’ and the Iranian threat. This webpage presents the perceived threat of Iran ‘under its current administration towards its own people, the peoples of the Middle East and the West in general’. ^37^ The webpage presents Iran as a major threat towards Israel, and argues that ‘it does not recognize the Holocaust, it openly calls for the annihilation of the state of Israel, it supports terrorist groups and it is actively pursuing a nuclear weapons program’. ^38^ The webpage further provides links to numerous articles in support of the view of Iran as an archenemy of Israel. Some titles are illustrative: ‘ *Iran behind rocket fire from Syria*’, ‘ *How Iran is arming Hezbollah*’, ‘ *Iran provided most of Hamas’ weapons*’, ‘ *Iran and Hezbollah support for Gaza terrorist organizations*’, etc. ^39^ This fixation with the Iranian threat of the Israeli ministry of foreign affairs website is indicative of the general perception of existential threats to Israel and Jewish identity in general. External threats – real or imaginary – have a cohesive function for the group and often silence internal opposition. The same is true for traumatic events – often more so than victorious events – since collective suffering (pogroms, expulsions, ethnic cleansing, genocide) functions cohesively for the group, authenticates its national identity and constitutes the ultimate verification of communal existence (i.e., we were persecuted because we were Jews – thus we exist as such) ( Koulos, 2016).

 Under ‘Foreign Policy’ we also find two more links that are relevant for this article, that is ‘Peace’ and ‘Terrorism’. Under ‘Peace’ the site offers a ‘guide to the Mideast process’ as well as about one hundred links to articles related to the peace process. These present the Israeli viewpoint on the peace process, arguing that ‘since its establishment in 1948, Israel has sought peace with its neighbors […] however, its efforts to reach out for peace and to open direct channels of dialogue were not met by similar efforts on the Arab side’. ^40^ Some article titles are also representative of the Israeli national narrative on the issue: ‘ *Negative Palestinian actions vs positive Israeli measures*’, ‘ *Jewish refugees from Arab and Muslim countries*’, ‘ *The dangers of a premature recognition of a Palestinian state*’, etc. ^41^ Illustrative of the Israeli national narrative is also an article titled ‘ *Israeli Settlements and International Law*’, which argues that ‘attempts to present Jewish settlement in West Bank territory (ancient Judea and Samaria) as illegal and "colonial" in nature ignores the complexity of this issue, the history of the land, and the unique legal circumstances of this case’. ^42^ The article further attempts to legally defend the Israeli position on the issue, concluding that ‘Jewish communities in this territory have existed from time immemorial and express the deep connection of the Jewish people to land which is the cradle of their civilization, as affirmed by the League of Nations Mandate for Palestine, and from which they, or their ancestors, were ousted’. ^43^ This summarizes the claim of the Jewish nation to Palestine that is based on a hereditary right, interpreted by some Jews on the basis of theologically inspired claims that the land was given/promised to them by God himself. Despite the mainstream Israeli national narrative on the issue of settlements however, the fact is that since the 1967 war the status of the occupied territories remains disputed and the Israeli settlements are not recognized by the international community ( Aron, 2019; Zertal & Eldar, 2009). At the same time, the Israeli project on the ‘Judaization’ of Palestine is for many a colonial project, as it is achieved through the dispossession of another people ( Zertal & Eldar, 2009; Zreik, 2016).

 Under the link ‘Terrorism’, the website offers about one hundred links to articles with information and updates on terrorist attacks against Israel and the Israeli response to these. It provides sub-links to ‘Operation Protective Edge’, ‘Palestinian Terror and Incitement’, ‘In Memoriam’, ‘Gaza Facts’, and ‘Hezbollah’. ^44^ The site provides further links and information on ‘Terror deaths in Israel: 1920–1999’, with number of fatalities per year; ‘Victims of Palestinian Violence and Terrorism since September 2000’, with exact date, name, age, origin, location and conditions of death for every victim; ‘Suicide and bombing attacks in Israel since 1993’, with date, location, number of victims and terrorist group responsible for every attack since 1993; and ‘In Memoriam’, with the names and date of death of the victims of ‘Palestinian violence and terrorism’ since 2000. This last one is a duplicate of the ‘Victims of Palestinian Violence and Terrorism since September 2000’ link and on the top of it, it has a picture of a lit candle. ^45^ We thus notice a digitalized national commemoration practice undertaken within the website of the ministry of foreign affairs of Israel itself. By detailing all the attacks against Israel, as well as the names and personal information of all the victims – the Jewish victims always – the website institutionalizes a kind of national commemoration service of the dead, who are identified with their names and are not mere numbers of the conflict. This way, the website partakes in the reproduction of the national Jewish narrative on the conflict and the symbolic reconstruction of the Jewish nation as a cyber community of suffering. The names of the dead enter the cyber pantheon of the Jewish glorious dead as in a form of time-capsule to be cyber-commemorated in perpetuity, digitally reproducing the mourning and suffering along with group cohesion that is usually strengthened through the common suffering. But what about the dead of the ‘other’ side? The number and the names of the Palestinian dead of this conflict do not seem to ‘belong’ to the Israeli cyberspace (even if they live in Israel or Israeli occupied territories). This is because as the Palestinians do not belong to the space of the Holy Land – according to Jewish nationalism – the same way they do not belong to its cyberspace.

 The final section of the website that is of interest for this article is the ‘About Israel’ section, which is mapped along ‘State’, ‘History’, ‘Among the Nations’, ‘Land’, ‘People’, ‘Culture’, ‘Economy’, ‘Science’, ‘Education’, ‘Health’, ‘Letter from Israel’, ‘Israel at 50’, ‘Israel in Maps’. ^46^ Under the ‘State’ there are links with information about the political structure, governance, the judiciary system, elections, the Knesset, the defence forces the presidency and the capital of the Israeli state. The webpage also provides pictures and detailed descriptions of the Israeli national flag and the Israeli national emblem. ^47^ Under ‘History’, the website offers sub-links with information on Israeli elections, Israeli wars, on Holocaust remembrance, on Popes’ visits to Israel and on Zionism. ^48^ Regarding the ‘Israel’s Wars’ webpage, this describes the military confrontations and operations of Israel since its establishment, praising the defence forces and cultivating a sense of national pride for the military triumphs of the nation. Wars in general tend to strengthen ethnocentrism and group cohesion ( Smith, 1981b), while their institutionalized commemoration (with parades, national days, or in this case with web articles) perpetuates these functions to future generations. In this sense the website serves Jewish national ideology, by commemorating and articulating the Israeli military activities. About Holocaust remembrance, the website provides more than one hundred thirty links to articles with information about Holocaust commemoration days and events since 1986. ^49^ With the Holocaust being the ultimate traumatic event for the Jews, it has become a symbolic component of Jewish national identity. As such, it functions cohesively and it provides a distinguishing element for the Jewish nation – being Jewish means, among others, that you are haunted by the Holocaust ( Koulos, 2016). Hence, its institutionalized commemoration is of utmost importance for the reproduction of Jewish national identity, and the website of the Israeli ministry of foreign affairs partakes in this endeavour. Finally, regarding the ‘Zionism’ part of this ‘History’ section, the site provides around thirty links to information about Zionism, the ideology, the movements, the leaders, its goals and a timeline of relevant to Zionism events. ^50^ Zionism is a form of Jewish nationalism, which advocated the migration of Jews to Palestine, in order to build their sovereign state ( Dieckhoff, 2017); it promoted a political modernization project, while drawing upon an ethnic identity preserved by religion ( Roshwald, 2004). Given the fact that the state of Israel was founded on the principles of Zionism ( Tekiner, 1991), by incorporating the links on Zionism the website of the ministry of foreign affairs reproduces online the national ideology that led to the establishment of Israel itself.

Another part of the website that is of interest to this article is regarding the land. There are two links under the ‘About Israel’ webpage that provide information about the land – the ‘Land’ and ‘Letter from Israel: the Land’. ^51^ Under the ‘Land’ link, there are further sub-links with information on the nature, the infrastructure, the urban and rural life, the climate and geography, and the rivers of Israel. ^52^ The introduction sub-link starts with ‘…a land flowing with milk and honey…(Exodus 3:8)’, ^53^ predisposing the readers of what they will read about: about the dream that kept the Jewish spirit and identity alive throughout the exile, the lost Eden that they found again – the Promised Land. The webpage further argues that

*‘Israel […] entered history some 35 centuries ago when the Jewish people forsook its nomadic way of life, settled in the Land and became a nation. Over the years, the Land was known by many names - Eretz Yisrael (Land of Israel); Zion, one of Jerusalem's hills which came to signify both the city and the Land of Israel as a whole; Palestine, derived from Philistia, and first used by the Romans; the Promised Land; and the Holy Land, to mention but a few. However, to most Israelis today, the country is simply Ha'aretz - the Land’*. ^54^

We notice here an attempt to apply a modern term – the term *nation* – to describe a past communal affiliation. Like the website of the Greek ministry of Foreign Affairs, the Israeli website adopts a primordialist view of the nation – that nations have existed since antiquity – disregarding modern factors in the emergence of the nations (bureaucratic state, capitalism, etc.). Another interesting point is the use of the word ‘land’ ( *Eretz*) instead of ‘territory’, which has political implications. In Hebrew, these words for land have biblical-historical origin and they are associated with the Zionist narrative that used them to connect the biblical story with national political goals. The word ‘territory’ ( *Shtachim*) on the other has no such connotations and usually refers to the occupied territories ( Aron, 2019). The webpage under the link ‘Letter from Israel’ again has information about the land of Israel – geography, infrastructure, flora and fauna, etc. The introduction of this part is quite interesting:

‘ *Israel, land of the Bible and the historic homeland of the Jewish people, is situated in the Middle East […] In this land, the Jewish people began to develop its distinctive religion and culture some 4,000 years ago, and here it has preserved an unbroken physical presence, for centuries as a sovereign state, at other times under foreign domination*’. ^55^

This part follows the main Jewish national narrative on the Jewish claim to the land, which is based on a hereditary right. This is interpreted mostly on the basis of theologically inspired claims that Jewish unity derives from the common descent of Jews from Abraham ( Tekiner, 1991). With the ‘unbroken physical presence’ of 4,000 years, the website – following the Jewish nationalist ideology – attempts to establish the element of continuity of the Jews in time and space. Continuity in time and over space is something that all modern nationalisms inculcate in order to ‘prove’ and ‘verify’ the ownership of a particular territory by their ethnic group. Continuity is an important element of the myth of spatial origins ( Smith, 1986), since space is a necessary dimension for a self-definition framework, and assumes special importance where claims to territory are being forwarded and contested. Spatial origins legitimate control over land, and assume an important role in controlling change by locating it in a distinctive area. No matter how drastic the change may be, it is always associated to a specific territory, a place that functions as a point of reference for the historical development, in a way that uprooted individuals are ‘restored’, if not physically at least symbolically, to ‘their’ homeland ( Smith, 1999). Hence, the claim of Jewish nationalism to the ‘unbroken’ continuity of Jews in the region for four millennia offers legitimacy of their claim over the space of the Holy Land. By reproducing this narrative online, Jewish nationalism enters the internet and claims as distinctively Jewish also the cyberspace of the Land.

 Finally, under the link ‘Israel in maps’ there are about 65 links to maps of Israel from topographic maps, to maps of the Kingdom of David and Solomon, to the Sykes-Picot agreement, to touristic maps of Jerusalem, to maps of war operations. ^56^ This detailed mapping enterprise of all aspects of Israeli national space through different historical periods functions as a documentation and ‘proof’ that the Land is Jewish. It is an attempt to Judaize the land by cartographically representing its Jewish history and topography only. By uploading all these maps on the Internet, the site delineates also the cyberspatial boundaries of Israel, stamping as distinctively ‘Jewish’ the Israeli cyberspace, leaving no space to alternative narratives or representations.

## Conclusions

This article has focused on the nationalization of cyberspace through state-led institutionalized practices. It has argued that the ideology of nationalism has shaped our perceptions of space and this has had implications in perceiving a new form of space – cyberspace – along national lines. The nationalization of space process – so successful in ideologically charging real spaces – managed to expand online to cyberspace. Cyberspace has become thus a field where nationalisms lay claims upon, flourish, are reproduced and frequently clash. Often, the nationalization of cyberspace is ‘assumed’ by independent, radicalised groups – like diasporas – with strong online presence, that propagate their ‘national’ interests online, and not necessarily connected to the official stances and positions of their respective governments.

 The article further analyzed the websites of the ministries of foreign affairs of four countries – Greece, the Netherlands, the USA and Israel – in order to examine how current state national ideology infiltrates cyberspace through them. All sites reproduce intrinsic elements as well as symbolic constituent components of their respective national identities online. The Greek website reflects the fixation of Greek nationalism with the past, the troubled relationship with the Greek significant ‘other’ – Turkey – and interprets the ‘national’ issues along Greek national lines. The Dutch website reproduces the anti-national, corporatist, tolerant and pluralistic traits of Dutch national identity, while the US website reflects the sense of mission and liberalism that have shaped American nationalism. Finally, the Israeli website echoes the Jewish national narrative regarding the claim to the land and propagates the Jewish national positions with regards to the conflict with the Palestinians. At the same time, the websites mostly ignore more cooperative, international and global agendas in favour of parochial, regional, and state-led ones that clearly reflect the respective foreign ministries' interests and visions. These agendas are relegated to the background insofar they do not fit the nationalist agenda. An example is the climate crisis, any mention of which is absent from the Dutch and Israeli websites, while it features as last of the ‘Global Issues’ of the Greek website. The US Department of State website seems to be the only one that is giving the issue the emphasis it deserves. The US website further refers to two more international issues that are absent from the other websites – the ‘Global Women’s Issues’ and ‘Ocean and Polar Affairs’. This is probably because of the self-view of the US as a World Power, compelled thus to deal with more ‘World’ Issues. Ignoring these crucial, global threats by most of the websites is indicative of the fact that the nationalization of cyberspace acts against the very interests, security and physical survival of nations.

 Thus, the digital avatars of the ministries of foreign affairs of these countries seem to align with the dictates of their official state-led national ideology. The foreign policy issues selected for diffusion need to fit their national agendas, while global emergencies are often deliberately ignored. In this way, national ideology infiltrates cyberspace, stamping it as distinctively national. The incorporation of cyberspace to the ‘national’ space seems to have been absolute since real space national practices – like war practices and national commemoration practices – are being adapted to cyber-spatial characteristics. The terminology already established – cyber-attacks, cyber-wars, cyber-defence, etc. – corresponds to the one used for real space national practices and seems to indicate the fact that cyberspace has embraced nationalism.

## Data availability

All data underlying the results are available as part of the article and no additional source data are required.
