# Supplementary material for: A digital territory to be appropriated: the state and the nationalization of cyberspace
Source: Open Res Eur. 2022 Mar 28;1:119. Originally published 2021 Oct 6. [Version 2] doi: 10.12688/openreseurope.14010.2 (PMC10446016; doi:10.12688/openreseurope.14010.2)
Supplement: Peer review history

# Peer review: Reviewer response for version 2

**DOI:** 10.21956/openreseurope.15824.r28950

**Published:** 2022-04-11

**Authors:** Tuba Eldem

**Affiliations:** 1 Department of Political Science and International Relations, Fenerbahce University, Istanbul, Turkey

This revised version has effectively addressed the points raised by the reviewer. It has further elaborated the conceptualization of cyberspace, unpacked the term of nationalization of cyberspace and clarified the aim of the paper in the methodology section. I hereby confirm that the revised version offers a significant contribution to the literature and is ready to pass peer review.

If applicable, is the statistical analysis and its interpretation appropriate?

Yes

Is the study design appropriate and is the work technically sound?

Partly

Is the work clearly and accurately presented and does it engage with the current literature?

Partly

Are the conclusions drawn adequately supported by the results?

Partly

Are sufficient details of methods and analysis provided to allow replication by others?

Yes

Are all the source data and materials underlying the results available?

Yes

Reviewer Expertise:

Global Cyberspace Governance and Security, Cyber Norms, International Cybersecurity,

I confirm that I have read this submission and believe that I have an appropriate level of expertise to confirm that it is of an acceptable scientific standard.

# Peer review: Reviewer response for version 2

**DOI:** 10.21956/openreseurope.15824.r28951

**Published:** 2022-04-05

**Authors:** Daniele Conversi

**Affiliations:** 1 Ikerbasque, Basque Foundation for Science, Department of Contemporary History, University of the Basque Country, Bilbao, Spain

Very clearly written, it is now enriched by new connections. The article is novel and will have a good impact on research.

If applicable, is the statistical analysis and its interpretation appropriate?

Yes

Is the study design appropriate and is the work technically sound?

Yes

Is the work clearly and accurately presented and does it engage with the current literature?

No

Are the conclusions drawn adequately supported by the results?

Yes

Are sufficient details of methods and analysis provided to allow replication by others?

Yes

Are all the source data and materials underlying the results available?

Yes

Reviewer Expertise:

NA

I confirm that I have read this submission and believe that I have an appropriate level of expertise to confirm that it is of an acceptable scientific standard.

# Peer review: Reviewer response for version 1

**DOI:** 10.21956/openreseurope.15098.r27762

**Published:** 2021-11-26

**Authors:** Daniele Conversi

**Affiliations:** 1 Ikerbasque, Basque Foundation for Science, Department of Contemporary History, University of the Basque Country, Bilbao, Spain

This is a well argued and objective analysis of the US, Greek, Dutch, and Israeli ministries of foreign affairs websites. It illustrates the way information is articulated, circulated and disseminated through official channels and networks. The use of content analysis is possibly adequate to the aims and goals of the article as it stresses the choice of specific sets of news against other news. This selective process is connected with the creation and appropriation of networks that the author adequately defines as "the nationalisation of the cyberspace".

 Some important aspects are, however, overlooked. Most important, could the author more clearly show how this process of news selection and information retrieval is filtered through those kinds of topics that aliment the ongoing process of splintering the Internet?

 Most importantly, I believe the author should emphasise how more cooperative, international and global agendas are ignored in favour of parochial, sectional, regional and state-led agendas that clearly reflect the respective foreign ministries' interests and visions as they are articulated in the analysed websites.

 Consequently, the author may need to ask how far crucial security threats, such as the climate emergency **,** are relegated to the background insofar as they do not fit the nationalist agenda.

 This is, in turn, linked to one question: what main foreign policy issues are selected for diffusion and why? The content analysis should also reveal how some of the websites deliberately ignore such global news and emergencies in favour of oblivion and, often, denial.

 The author mentions national control of the recognised maritime space: In fact, the United Nations’ Law of the Sea Convention (UNCLOS) for the management of marine natural resources should be connected to the way nationalism is unable to tackle global issues, such as biodiversity loss. The very way that natural gas and oil reserves discovered in the respective continental shelves are rebranded is more indicative of the presence of ‘resource nationalism’ - a term that should be better linked to the notion of "nationalization of cyberspace".

 On the other hand, the author presents good evidence in the way the four ministries' websites are nationalistically captured resulting in a "nationalization of cyberspace" against the original cosmopolitan dream of the Internet as a neutral arena of international communications **.  **Accordingly, the four foreign ministries are active promoters and prominent actors in the broader propagation of the splintering of the cyberspace into tightly bound national areas.

 But this should be understood in a very broad sense, both as the propagation of ‘hot’ nationalism in regions characterised by persisting and deeply rooted national conflicts and as forms of "banal nationalism" in more ordinary everyday settings (as described by Michael Billig 1995).

 Also, the notion of *cyberspace* needs to be situated with more precision according to which components or elements are privileged in each case. I think this could be resolved by adding a few sentences that could clarify the conceptual reach of the term and identify its components. Among the layers that needs to be added are autonomous forces, such as those arising in the diasporas in the process elsewhere identified as “irresponsible radicalisation”. By briefly considering this important aspect beyond official websites, the evidence of the ongoing nationalization process could be more nuanced and further clarified. Beyond official government and ministries websites, Facebook’s role in inciting or assisting nationalist violence has also been studied in recent research.

 In conclusion, while the notion of “nationalization of cyberspace” is very promising, it needs to be supplemented by a short discussion about the broader process of autonomous radicalisation, which within the diasporas does not appear to be necessarily connected to the propaganda efforts of the ministries of foreign affairs. The author also needs to consider how crucial security threats at the global level such as climate change have been often ignored in most of these official websites at least until recently. This could clarify how the "nationalisation of cyberspace" acts in practice against the very security, interests, and survival of nations.

 Minor revisions required.

If applicable, is the statistical analysis and its interpretation appropriate?

Yes

Is the study design appropriate and is the work technically sound?

Yes

Is the work clearly and accurately presented and does it engage with the current literature?

No

Are the conclusions drawn adequately supported by the results?

Yes

Are sufficient details of methods and analysis provided to allow replication by others?

Yes

Are all the source data and materials underlying the results available?

Yes

Reviewer Expertise:

Nationalism and the Internet Long distance nationalism

I confirm that I have read this submission and believe that I have an appropriate level of expertise to confirm that it is of an acceptable scientific standard, however I have significant reservations, as outlined above.

## References

[1] : Banal Nationalism. *Sage Publishing* .1995;

# Response

**Published:** 2022-03-20

**Authors:** Thanos Koulos

**Affiliations:** 205812, The Netherlands

Thank you very much for your review. Your points have been taken into consideration, discussed and addressed in 'the naitonalization of all spaces' and 'conclusions' sections.

# Peer review: Reviewer response for version 1

**DOI:** 10.21956/openreseurope.15098.r27760

**Published:** 2021-11-11

**Authors:** Tuba Eldem

**Affiliations:** 1 Department of Political Science and International Relations, Fenerbahce University, Istanbul, Turkey

This article examines websites of Greek, Dutch, US and Israeli ministries of foreign affairs through content analysis. It outlines how the websites are structured, how the missions and visions of foreign ministries are defined and what the main foreign policy issues are. More importantly, through the content analysis of the four foreign ministry websites, the author unpacks how each official state website constructs and propagates its own nationalist ideology. These sections are informative and well-organized.

 I have, however, concerns over the conceptual framework, and thus methodology. The author presents the use of national ideology in the official state websites as evidence of “nationalization of cyberspace” and argues that this “contradicts the original purpose of the Internet as a liberating, post-national medium, offering new forms of community”.

 The conceptualization and measurement of nationalization of cyberspace needs to be well-situated in the literature and better developed. To be more specific, can the propagation of the national ideology in the institutional websites of foreign ministries be really considered as ‘nationalization of cyberspace’?  After all, the concept of cyberspace is a term broader than Internet. It includes physical, logical and cyber-persona layers. Physical network refers to the machines – such as the computers, routers, cables, servers, cellphone towers, and satellites – that establish the mechanical and electrical, magnetic, and optical lines of communication. Logical network refers to the logical instructions and software that operate communications traffic, such as domain name system (DNS) and Internet service providers (ISP). And finally cyber-persona or social layer refers to the sphere through which videos, images, sounds, and texts circulate.

 When author refers to the nationalization of cyberspace, which layer does he refer to? Since the article only focuses on the official websites, it seems to refer to social layer or what in the military realm is referred to as strategic communications. But even within this limited layer, the evidence provided fails to denote nationalization of strategic communication/social/cyber-persona layer. In order to arrive at such a conclusion, one needs to see further evidence of nationalization attempts, such as a concerted effort in social media networks that propagate same or similar ideas or positions outlined in the foreign ministry websites, use of computational propaganda or nation-specific information control policies that are in line with the national ideology dispersed in the official websites such as filtering, surveillance, legal regulations regulating the dissemination of information online, content control, etc.

 In the literature, nationalization of cyberspace generally refers to a shift from laissez-faire and market-oriented approaches towards the state-directed controls and regulations. Although such a trend is also visible in Western liberal democracies, it is more prevalent among those cyber-sovereignist states such as China and Russia, which try to eradicate the borderless nature of cyberspace by controlling all of these three layers through constructing underwater communication networks, various forms of information control and data localization policies as well as attempting to shape international cyberspace governance regimes. Nationalization of cyberspace thus refers more of demarcating new boundaries in national cyberspace generally for security purposes through implementing  cyberspace regulations, internet policies, cyber laws and other  regulatory frameworks, thereby giving rise to a ‘splinternet’.

 I therefore consider that the use of “nationalization of cyberspace” as a central conceptual framework may not be appropriate. I suggest reframing the article centering on the reproduction of nationalism or national identities through governmental websites, rather than nationalization of cyberspace.

If applicable, is the statistical analysis and its interpretation appropriate?

Yes

Is the study design appropriate and is the work technically sound?

Partly

Is the work clearly and accurately presented and does it engage with the current literature?

Partly

Are the conclusions drawn adequately supported by the results?

Partly

Are sufficient details of methods and analysis provided to allow replication by others?

Yes

Are all the source data and materials underlying the results available?

Yes

Reviewer Expertise:

Global Cyberspace Governance and Security, Cyber Norms, International Cybersecurity,

I confirm that I have read this submission and believe that I have an appropriate level of expertise to confirm that it is of an acceptable scientific standard, however I have significant reservations, as outlined above.

# Response

**Published:** 2022-03-20

**Authors:** Thanos Koulos

**Affiliations:** 205812, The Netherlands

Thank you very much for your review. The paper defines the nationalization of cyberspace as the ideological charging of the cyber-footprint of the nation (abstract), while states that it focuses on the official websites of the ministries of foreign affairs and how they reproduce the nationalist ideology (Methodology). See also points of 2 ^nd^ reviewer. At the same time, it addresses raised points and defines cyberspace under ‘the nationalization of all spaces’ section.
